# The regulatory network of ThbZIP1 in response to abscisic acid treatment

**DOI:** 10.3389/fpls.2015.00025

**Published:** 2015-02-10

**Authors:** Xiaoyu Ji, Guifeng Liu, Yujia Liu, Xianguang Nie, Lei Zheng, Yucheng Wang

**Affiliations:** ^1^Key Laboratory of Biogeography and Bioresource in Arid Land, Xinjiang Institute of Ecology and Geography, Chinese Academy of SciencesUrumqi, China; ^2^State Key Laboratory of Tree Genetics and Breeding, Northeast Forestry UniversityHarbin, China; ^3^College of Food Engineering, Harbin University of CommerceHarbin, China

**Keywords:** bZIP transcription factor, cis-acting element, abiotic stress, yeast one hybrid, gene expression regulation, microarray analysis

## Abstract

Previously, a bZIP transcription factor from *Tamarix hispida*, ThbZIP1, was characterized: plants overexpressing ThbZIP1 displayed improved salt stress tolerance but were sensitive to abscisic acid (ABA). In the current study, we further characterized the regulatory network of ThbZIP1 and the mechanism of ABA sensitivity mediated by ThbZIP1. An ABF transcription factor from *T. hispida*, ThABF1, directly regulates the expression of ThbZIP1. Microarray analysis identified 1662 and 1609 genes that were respectively significantly upregulated or downregulated by ThbZIP1 when exposed to ABA. Gene ontology (GO) analysis showed that the processes including “response to stimulus,” “catalytic activity,” “binding function,” and “metabolic process” were highly altered in ThbZIP1 expressing plants exposed to ABA. The gene expression in ThbZIP1 transformed plants were compared between exposed to ABA and salt on the genome scale. Genes differentially regulated by both salt and ABA treatment only accounted for 9.75% of total differentially regulated genes. GO analysis showed that structural molecule activity, organelle part, membrane-enclosed lumen, reproduction, and reproductive process are enhanced by ABA but inhibited by salt stress. Conversely, immune system and multi-organism process were improved by salt but inhibited by ABA. Transcription regulator activity, enzyme regulator activity, and developmental process were significantly altered by ABA but were not affected by salt stress. Our study provides insights into how ThbZIP1 mediates ABA and salt stress response at the molecular level.

## Introduction

The basic leucine zipper (bZIP) transcription factor family is one of the largest and most diverse in plants (Nijhawan et al., 2008). bZIP proteins play important roles in many biological processes. In plants, the bZIP family has been identified comprehensively or predicted in several plant genomes. For instance, seventy-five bZIP genes have been identified in Arabidopsis (*Arabidopsis thaliana*) (Jakoby et al., 2002), 89 in rice (*Oryza sativa*) (Nijhawan et al., 2008), 92 in Sorghum (Wang et al., 2011), and 125 in maize (*Zea mays*) (Wei et al., 2012). Plant bZIPs are involved in many metabolic processes, such as energy metabolism (Baena-González et al., 2007), as well as in the development of many organs and tissues, including seed maturation and germination (Izawa et al., 1994; Toh et al., 2012), floral induction and development (Chuang et al., 1999; Walsh and Freeling, 1999; Strathmann et al., 2001; Abe et al., 2005; Muszynski et al., 2006; Gibalova et al., 2009; Iven et al., 2010), embryogenesis (Shiota et al., 2008; Guan et al., 2009), photomorphogenesis (Holm et al., 2002; Huang et al., 2012) and senescence (Smykowski et al., 2010). In addition, they are also involved in responses to a variety of abiotic/biotic stimuli, such as high salinity (Hsieh et al., 2010), drought (Yoshida et al., 2010; Chen et al., 2012), cold stress (Shimizu et al., 2005; Liu et al., 2012a), heat stress (Liu et al., 2012b), pathogen infection (Thurow et al., 2005) and hormone signaling, such as abscisic acid (ABA) (Choi et al., 2000; Lopez-Molina et al., 2002; Yoshida et al., 2010), ethylene (Zander et al., 2012) and light signaling (Zhou et al., 2014).

ABA is an important phytohormone that plays pivotal roles in the regulation of various processes and responses, such as seed development and germination, root and stem growth, and biotic and abiotic responses (Busk and Pagès, 1998; Leung and Giraudat, 1998). When plants are confronted with abiotic stresses, such as salt, drought or cold, at least two independent signal transduction pathways are triggered: the ABA-dependent and ABA-independent signaling cascades (Shinozaki and Yamaguchi-Shinozaki, 1996). Abscisic acid-responsive element (ABRE)-binding proteins play important roles in ABA-dependent signaling pathway. The bZIPs are typical ABRE-binding transcription factors, which bind to ABRE elements and trans-activate downstream gene expression (Yamaguchi-Shinozaki and Shinozaki, 2005).

The genus *Tamarix* (tamarisk, salt cedar) is a woody halophyte, which is shrub or small tree, and is widely distributed in the saline soils or drought-stricken areas of Central Asia. *Tamarix hispida*, a specie of genus *Tamarix*, is highly tolerant to salinity, drought and high temperature, indicating that it has efficient abiotic stress defense systems. Therefore, *T. hispida* is a suitable model in which to characterize genes and mechanisms responsible for stress tolerance in plants. Previously, we showed that the *Tamarix hispida* ThbZIP1 gene product specifically binds to C-box, G-box, and A-box motifs to regulate serial stress-related genes. Transgenic Arabidopsis plants overexpressing ThbZIP1 exhibited an enhanced tolerance to drought and salt, but were sensitive to ABA (Ji et al., 2013). In the present study, to investigate the expression regulatory network of ThbZIP1 in response to ABA, the upstream regulator of ThbZIP1 was identified, and the responses of plants overexpressing ThbZIP1 sensitive to ABA stress were analyzed using a microarray. Furthermore, the gene expression profiles regulated by ThbZIP1 in response to salt and ABA were compared. A regulatory working model was proposed to show the function of ThbZIP1 in response to ABA. These results may be helpful to reveal the bZIP regulation network in response to abiotic stress.

## Materials and methods

### Plant materials and growth conditions

Seedlings of *Tamarix hispida* were grown in pots containing a mixture of turf peat and sand (2:1 v/v) in a greenhouse under controlled conditions of 70% relative humidity, light/dark cycles of 14/10 h, and 24°C. To induce abiotic stresses, three-month-old *T. hispida* seedlings were watered on their roots with a solution of 0.4 M NaCl, 20% (w/v) PEG6000, 100 μM ABA, or 50 μM MV (methylviologen) for 3, 6, 9, 12 or 24 h. For cold treatments, three-month-old seedlings were placed in an incubator at 4°C (light/dark cycles of 14/10 h) for 3, 6, 9, 12 or 24 h. After each stress treatment, at least 20 seedlings were harvested and pooled for subsequent study.

Seeds of *Arabidopsis thaliana* were surface sterilized and plated on a 1/2 Murashige-Skoog (MS) solid medium supplemented with 1% sucrose. One week-old seedlings were then transferred into pots filled with the perlite/soil mixture in a growth chamber (16 h 180 μmolm^−2^s^−1^ light/8 h dark photoperiod at 22°C). Four-week-old seedlings were watered on their roots with a solution of 150 mM NaCl or 10 μM ABA for 3 h, and were used as experimental materials. The harvested seedlings in each treatment were divided into three portions as independent biological replicates.

### Analysis of the upstream regulators of ThbZIP1

Previously, we constructed seven transcriptomes of *T. hispida* and clustered unigenes from these transcriptomes. The transcription factors (TFs) from different families were identified from the unigenes, PCR amplified and cloned into pGADT7-Rec2 (Clontech) to form a cDNA library (designed as TFs library) for a yeast one-hybrid assay. Three tandem copies an ABRE motif sequence were cloned into a pHIS2 vector (designed as pHIS2-ABRE, see Supplementary Table S2 for the primers used), and screened with TFs library in a one-hybrid assay (Clontech, Palo Alto, CA, USA). The interactions of p53HIS2 (three tandem copies of the cis-acting DNA consensus sequence inserted into the multiple cloning site (MCS) of pHIS2, which is recognized by p53) with the tested TFs were used as negative controls.

An ABF (ThABF1 GenBank number: JX169810) was identified that bound to the ABRE motif. Subsequently, the ABRE core motif, “ACGTG,” was mutated to “CCGTG,” “ACGCA,” and “CAACA” (designed as pHIS2-A-M1, -A-M2, -A-M3, respectively; see Supplementary Table S2 for primers used), and cloned into pHIS2. The interactions of ThABF1 with the ABRE motif and its mutants were studied using Yeast one-hybrid analysis to determine if ThABF1 could bind to the promoter of ThbZIP1 by interacting with the ABRE motifs, pHIS2 constructs that harbored the truncated promoter of ThbZIP1 containing the ABRE (pHIS2-proA(+)) or lacking the ABRE (pHIS2-proA(−)) were generated as reporter vectors (see Supplementary Table S2 for the primers used). Their interactions with ThABF1 were studied using yeast one-hybrid analysis.

### Transient expression analysis

**Figure 1:**
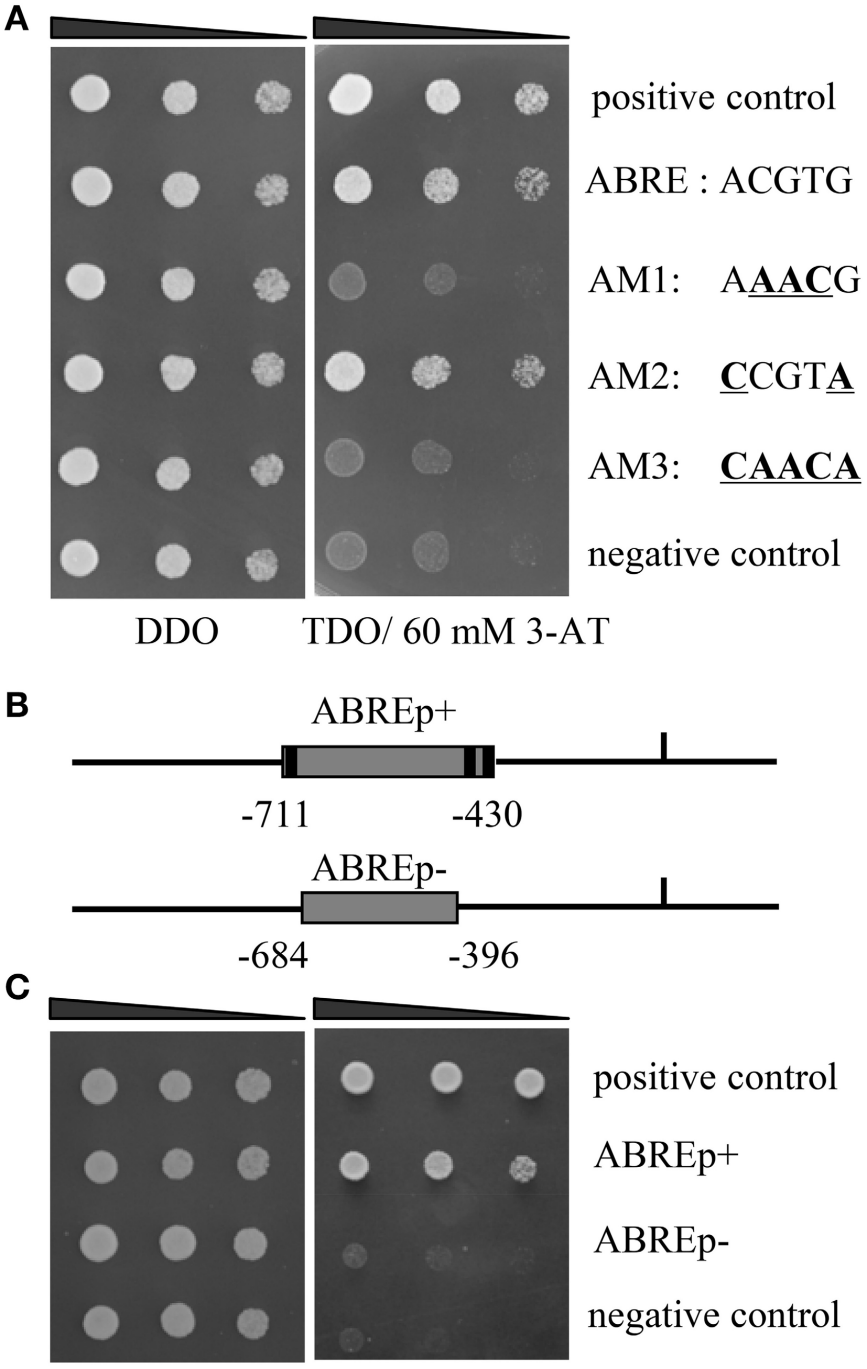
**Identification of the upstream regulators of ThbZIP1. (A)** The bindings of ThABF1 to the ABRE motifs and the mutated ABRE sequences. p53HIS2/pGADT7-p53 and p53HIS2/pGADT7- ThABF1 were used as positive and negative controls, respectively. Positive transformants were identified by spotting serial dilutions (1/1, 1/10, 1/100) of yeast onto SD/-His/-Leu/-Trp (TDO) plates. The transformants grown at SD/-Leu/-Trp (DDO) were used as positive controls for transformant growth. **(B)** Schematic diagram of the truncated ThbZIP1 promoter used in the Y1H analysis. ABREp+ or ABREp− indicates the truncated promoter of ThbZIP1 containing three ABRE motifs or lacking these ABRE motifs. **(C)** Y1H analyses of the specific binding of ThABF1 to the truncated ThbZIP1 promoter containing or lacking the ABRE motifs. pHIS-ABREp+, pHIS-ABREp−:pHIS2 reporter vector harboring one copy of the truncated promoter containing three ABRE motifs (pHIS-ABREp+) or without ABRE motifs (pHIS-ABREp−).

To further verify these interactions, the three tandem copies of the ABRE motif and its mutant A-M3 (CAACA) were fused separately to the minimal 35S promoter (−46 to +1) to drive GUS in a reformed pCAMBIA1301 (in which the 35S::Hygromycin region was deleted), and designed as vectors pCAM-ABRE and pCAM-A-M3, respectively (see Supplementary Table S3 for the primers used). The promoter fragments of ThbZIP1, which contained ABRE motifs, or lacked ABRE motifs (shown in Figure 1B), were fused separately to the minimal 35S promoter (−46 to +1) to substitute its 35S promoter to drive GUS in a reformed pCAMBIA1301 as reporter vectors. The reformed pCAMBIA1301 construct (35S::hygromycin had been deleted, and a 46 bp minimal promoter was inserted between the region of BglII site and ATG of GUS) that harbored the promoter fragment with ABRE motifs was named as pCAM-ABREp+, and the promoter fragment lacking the ABRE motif was named as pCAM-ABREp− (see Supplementary Table S3 for the primers used). The effector vector was constructed by cloning the full open reading frame (ORF) of the ThABF1 gene into pROKII driven by the 35S promoter (named as pROKII-ThABF1). Both of the reporter vectors and their corresponding effector vectors were co-transformed into tobacco leaves using the particle bombardment method (Bio-Rad, Hercules, CA, USA), following the manufacturer's instructions for the Biolistic® PDS-1000/He Particle Delivery System (Bio-Rad). The transformation conditions were 9 cm target distance, 1100 psi helium pressure, and two bombardments. The quantity ratio of reporter, effector, and 35S::Luc were 2:2:1. Three independent biological repeats were performed. Transformation with the reporter plasmids or effect plasmids alone served used as negative controls. The transformation of pCAMBIA1301 alone (CaMV35S) was used as a positive control. To normalize the transformation efficiency, the construct harboring a luciferase gene driven by the CaMV 35S promoter (35S::Luc) was also cotransferred. GUS histochemical staining was performed as described by Jefferson (Jefferson, 1989), and the GUS activity levels were determined according to the method of Jefferson (Jefferson et al., 1987).

### Chromatin immunoprecipitation (ChIP) analysis

To further confirm the binding of ThABF1 to the promoter of ThbZIP1, ChIP analysis was performed. The coding regions of ThABF1 without the termination codon were ligated in frame to the N-terminus of GFP driven by the CaMV 35S promoter to generate a ThABF1::GFP fusion gene, which was transformed into *T. hispida* plants using the transient transformation system (Ji et al., 2013). Briefly, protein and DNA were cross-linked using 3% formaldehyde. The purified cross-linked nuclei were sonicated to shear the chromatin into 0.5–1 kb fragments. We saved 1/10 volume as an input control. The remaining sonicated chromatin was incubated with an anti-GFP antibody (Beyotime, Shanghai, China). The antibody-bound complex was precipitated with protein A+G agarose beads (Beyotime). The DNA fragments were released from the immunoprecipitated complexes by reversing the cross-linking at 65°C for 3 h. Immunoprecipitated DNA was purified by chloroform extraction. PCR was performed and visualized by gel electrophoresis. The primers used for PCR are shown in Supplementary Table S4. PCR was performed as follows: 94°C for 2 min; 35 cycles of 94°C for 15 s, 58°C for 30 s, and 72°C for 30 s; and 72°C for 5 min. 35S::GFP transgenic plantswere used as a parallel control for ChIP experiment. Real-time PCR was performed using SYBR Green Real-time PCR Master Mix (Toyobo, Osaka, Japan) in a 20 μl reaction volume on a MJ Research OpticonTM2 instrument (Bio-Rad). The PCR cycling parameters was as follows: 94°C for 2 min; 45 cycles of 94°C for 20 s, 56°C for 30 s, and 72°C for 1 min; and 80°C for 1 s for plate reading. The sequence of Actin (FJ618517) was used as the internal control. Primers are listed in Supplementary Table S4. The ChIP assays were performed three times, with similar results, and data are mean ± SD from three independent experiments.

### Real-time RT-PCR analysis of gene expression

To investigate the expression of ThbZIP1 and ThABF1 in response to different biotic stresses, real-time RT-PCR were performed using Actin (FJ618517) α-tubulin (FJ618518) and β-tubulin (FJ618519) as internal controls (see Supplementary Table S5 for the primers used). PCR was performed on a MJ Research OpticonTM2 instrument with the following conditions: 94°C for 30 s; 45 cycles of 94°C for 12 s, 58°C for 30 s, 72°C for 40 s; and 80°C for 1 s for plate reading. The relative expression levels of the products were calculated according to the 2^−ΔΔCt^ method (Pfaffl et al., 2002).

### Microarray experiments and data analysis

Four-week-old seedlings of wild-type Col-0 and ThbZIP1 transgenic plants without treatment, or subjected to ABA treatment for 3 h, were used for microarray analyses using an Agilent Arabidopsis oligonucleotide microarray; two biological replications were performed. Two microgram aliquots of total RNA from ABA-treated plants were prepared and hybridized to the microarray using the Gene-Chip® 3′IVT Express Kit (Agilent) and Gene-Chip® hybridization. After hybridization, the microarray slides were washed and stained according to the manufacturer's standard protocol (Agilent). Normalization of all arrays was performed by quantitative normalization using MAS 5.0 to standardize the distribution of probe intensities for each array in a set of arrays (Jia et al., 2012; Wang et al., 2012). The differentially expressed genes (up- or downregulated) between the NaCl-stressed and ABA-stressed plants were selected with a significance *P*-value of <0.05 and analyzed using Welch's *t*-test (Wolfinger et al., 2001). Gene ontology (GO) terms for the differentially expressed genes under ABA treatment were divided into three levels: molecular function, cellular component, and biological process. The gene expression data set was deposited at the Gene Expression Omnibus database (GSE62888).

To verify the microarray data, the confirmed differentially expressed genes were randomly selected for real-time PCR analysis. The genes and primers used for the RT-PCR assays are listed in Supplementary Table S6. Amplification conditions were as follows: hot start at 94°C for 30 s; followed by 45 cycles of 12 s at 94°C, 30 s at 60°C, 40 s at 70°C; and 1 s at 80°C for plate reading. The Arabidopsis α-tubulin (XM_002301092) and actin 3 (XM_002308329) genes were used as the internal controls; three biological repeats were performed. The relative expression levels of the products were calculated according to the 2^−ΔΔCt^ method (Pfaffl et al., 2002).

### Searching for ThbZIP1-binding motifs in gene promoters

Thirty-three genes differentially regulated by ThbZIP1 under ABA stress conditions were randomly selected to search for ThbZIP1-binding sequences in their promoters region. The promoter sequences (from −1 to −1000) of these genes were derived from TAIR database (http://www.arabidopsis.org/). For identification of ThbZIP1-binding motifs, the sequences of C-, G- and A-boxes were searched in the promoter regions of these genes.

### Statistical analyses

Statistical analyses were carried out using SPSS 16.0 (SPSSInc, Chicago, IL, USA) software. Data were compared using Student's *t*-test. Differences were considered to be significant if *P* < 0.05. ^*^ represented 0.01 < *P* < 0.05.

## Results

### Results and discussion

#### The ThABF1 is the upstream regulator of ThbZIP1

Previously, the 1571 bp promoter of ThbZIP1 was cloned using a genome walking kit (Takara, Dalian, China) (Ji et al., 2013). The cis-acting elements in the promoter of ThbZIP1 were identified, including ABRE, DOFCOREZM, MYBCORE, W-boxes, and E-boxes (Supplementary Figure S1). There were three ABRE (“ACGTG”) motifs in the promoter of ThbZIP1 (Figure 1), indicating that ABRE motifs may play important roles in the regulation of ThbZIP1 expression. To study which TF could bind to the ABREs to regulate the expression of ThbZIP1, yeast one hybrid (Y1H) analysis was performed. The result showed that an ABF (ThABF1) could bind to the ABRE motifs (Figure 1A). To further determine the specificity of the binding of ThABF1 to the ABRE motifs, the ABRE motifs were mutated (Figure 1A), and the interactions between ThABF1 and the mutated motifs were investigated using Y1H analysis. The results showed that ThABF1 failed to interact with all the mutated ABRE motifs (Figure 1A), indicating the binding of ThABF1 to the ABRE motifs is specific.

To further determine whether ThABF1 can bind to the promoter of ThbZIP1 by binding to ABRE motifs in the promoter, the interactions of a truncated ThbZIP1 promoter containing ABRE motifs (pHIS2-ABREp(+)) or lacking ABRE motifs (pHIS2-ABREp(−)) (Figure 1B) with ThABF1 was investigated using Y1H analysis. The results showed that ThABF1 could bind to the truncated promoter with ABRE motifs, but failed to bind to the truncated promoters lacking ABRE motifs (Figure 1C), indicating that ThABF1 can interact with the promoter of ThbZIP1 via binding to the ABRE motifs.

#### The interaction between ThABF1 and ABRE motifs in tobacco plants

**Figure 2:**
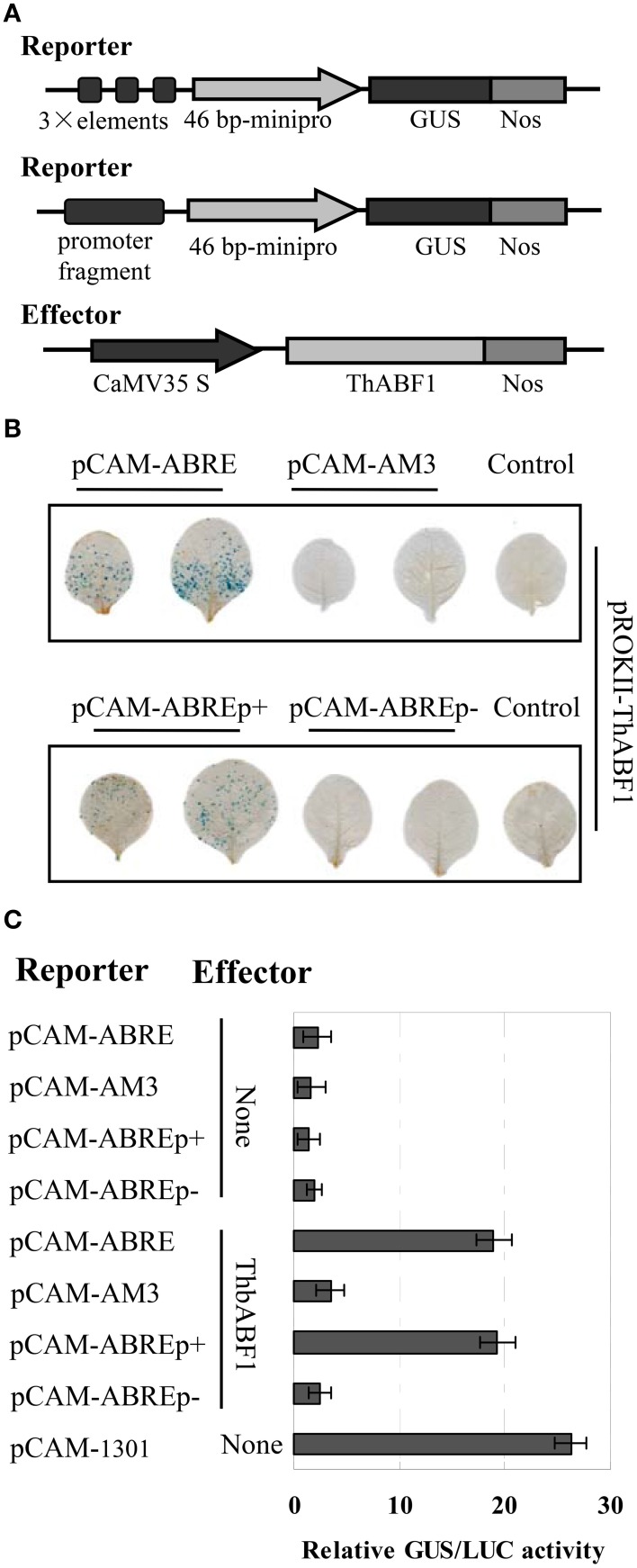
**Analysis of the interaction of ThbZIP1 with the ABRE motif in plants**. **(A)** Schematic diagram of the reporter and effector constructs. **(B)** The coexpression of the reporter and effector vectors in tobacco leaves. **(C)** GUS activity assay of the binding of ThABF1 to the ABRE motif and the ThbZIP1 promoter in tobacco plants. pCAM-1301: transformation of the reformed empty pCAMBIA1301 (in which the 35S::Hygromycin region was deleted) as a positive control; None: cotransformation of empty pROKII and the reporters. The transformation efficiencies were normalized using LUC activity. Data represent mean values of three independent experiments.

To further confirm the interaction between ThABF1 and the ABRE motifs, the effector construct (pROKII-ThABF1) (Figure 2A) was co-transformed into tobacco together with its corresponding reporter plasmids pCAM-ABRE (*GUS* gene under control of three tandem copies of ABRE), pCAM-AM3 (*GUS* gene under control of three tandem copies of mutant ABRE), pCAM-ABREp+ (*GUS* gene under control of the truncated promoter containing ABREs), pCAM-ABREp− (*GUS* gene under control of the truncated promoter lacking ABREs) (Figure 2A). Histochemical staining and GUS activity assay both showed that the *GUS* reporter gene was activated when pROKII-ThABF1 was introduced together with pCAM-ABRE or pCAM-ABREp+ (Figures 2B,C). However, the co-transformation of pCAM-ABREp− and pROKII-ThABF1 and transformation of reporter construct alone failed to activate GUS (Figures 2B,C). These data indicated that the ThABF1 protein binds to the ABRE motifs in tobacco plants.

#### The binding of ThABF1 to the promoter of ThbZIP1 occurs in tamarix hispida

To further study whether the binding of ThABF1 to the promoter of ThbZIP1 actually occurs in *T. hispida*, ChIP analysis was performed. We carried out ChIP using transgenic *T. hispida* plants transformed with the 35S::ThABF1::GFP fusion gene; *T. hispida* plants transformed with 35S::GFP were used as negative control.

**Figure 3:**
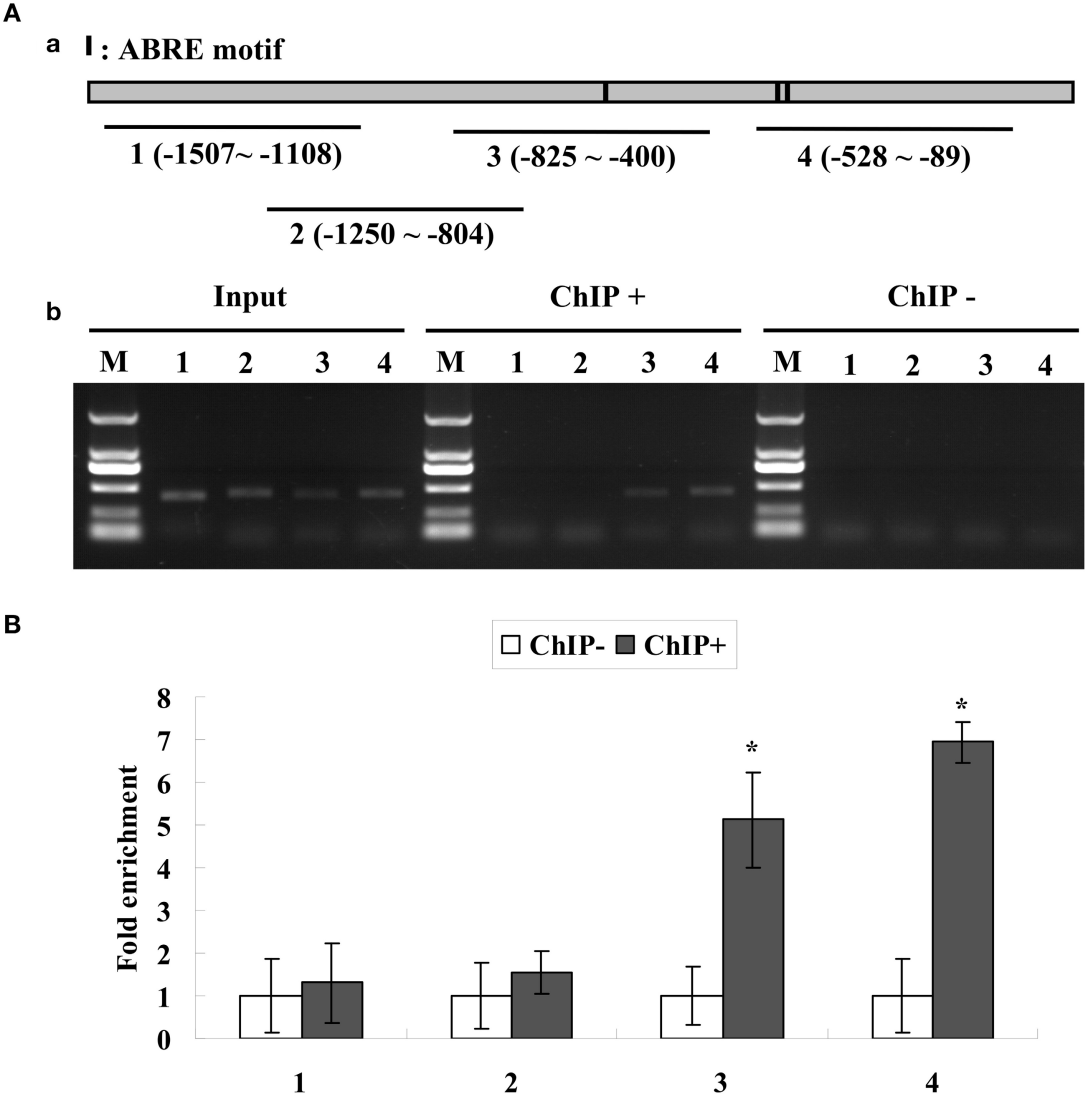
**(A)** ChIP analysis of the binding of *ThABF1* to the promoter of *ThbZIP1*. Black lines represent the *ThbZIP1* promoter region and black shadings represent the ABRE motifs. Input, PCR amplification of sonicated chromatin; ChIP+: PCR amplification of chromatin immunoprecipitated from 35S::*ThABF1*; ChIP−: PCR amplification of chromatin immunoprecipitated from the HA antibody (negative control). M, Marker DL2000. 1–4: PCR products of *ThbZIP1* promoter fragments using four pairs of primers. **(B)** ChIP quantitative PCR assay for the bindings of *ThABF1* to ABRE motifs. The enrichment of each truncated promoter was analyzed by real-time PCR. After normalization against *Actin*, the values in ChIP− were designed as 1 for qRT-PCR analysis. Asterisks represent the fragments showing significant enrichment by PCR production (*P* < 0.05); Input, Input DNA (positive controls); CHIP+: chromatin immunoprecipitated with anti-GFP antibody; CHIP−: chromatin immunoprecipitated with HA antibody (negative controls).

The results showed that the truncated promoters of ThbZIP1 could be amplified from input and chromatin DNA of 35S::ThABF1::GFP transgenic plants immunoprecipitated with GFP antibody (ChIP+); however, the chromatin DNA of 35S:GFP transgenic plants immunoprecipitated with GFP antibody (ChIP−) failed to amplify the corresponding PCR products (Figure 3A). Consistent with the results of gel electrophoresis, real-time PCR analysis showed that the promoter fragments were significantly enriched in the immunoprecipitated chromatin of 35S::ThABF1::GFP transgenic plants, but were not enriched in the immunoprecipitated chromatin of 35S::GFP plants (Figure 3B). These results indicated that the binding of ThABF1 to the promoter of ThbZIP1 actually occurs in *T. hispida*. Taken together, these results suggested that ThABF1 is one of the upstream regulators of ThbZIP1.

#### The expressions of both ThbZIP1 and ThABF1 in response to abiotic stresses

**Figure 4:**
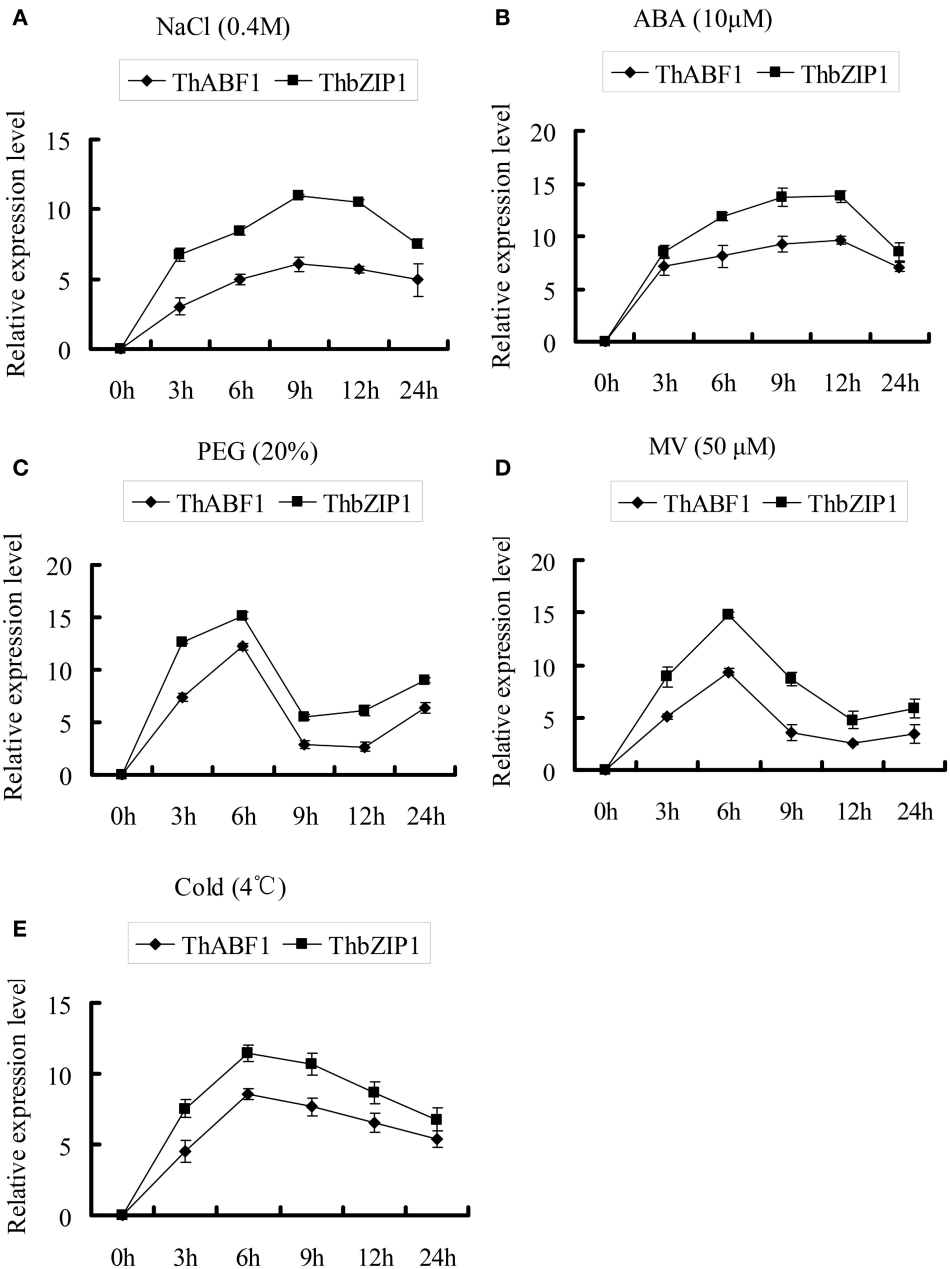
**The expression patterns of *ThABF1* and *ThbZIP1* in response to salt (A), ABA (B), osmotic (C), oxidative (D) and cold (E)**. The relative expression level = log_2_ (transcription level under stress treatment/transcription level under control condition). The error bars were obtained from multiple replicates of the real-time PCR.

Previously, we studied the expression of ThbZIP1, and the results showed that it can be highly induced by NaCl, PEG6000, ABA, MV, and cold treatments (Ji et al., 2013). In the present study, we studied the expression of ThABF1 in response to these abiotic stresses. The results showed that NaCl, PEG6000, ABA, MV, and cold treatments also induced ThABF1 (Figure 4). In addition, ThbZIP1 and ThABF1 shared very similar expression patterns in response to NaCl, PEG6000, ABA, MV, and cold, which further suggested that they are involved in a common regulatory cascade.

#### Genes regulated by ThbZIP1 when exposed to ABA

Previously, we generated the transgenic tobacco and Arabidopsis plants overexpression of *ThbZIP1*. The studies showed that *ThbZIP1* transformed plants showed improved salt and drought stress tolerance, seed germination rate were significantly enhanced, and fresh weight gain and root growth were increased compared with WT plants did under salt and drought stress condition (Wang et al., 2010; Ji et al., 2013). On the contrary, *ThbZIP1* transformed plants showed significantly decreased seed germination rate, fresh weight gain, and root growth compared with WT when exposed to ABA treatment condition, indicating that plants expressing *ThbZIP1* exhibit increased sensitivity to ABA treatment (Ji et al., 2013).

**Figure 5:**
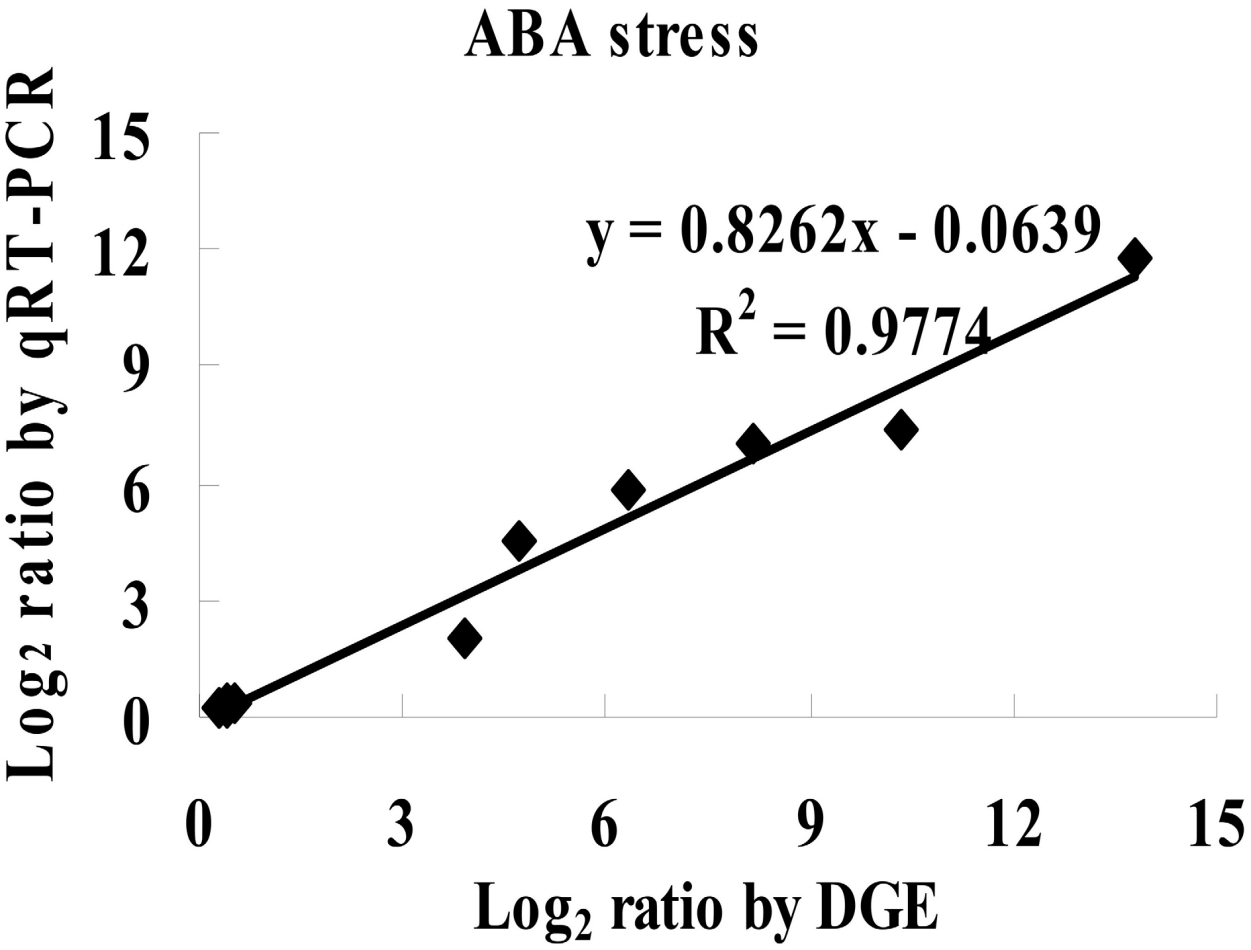
**Comparison of the results of microarray and real-time RT-PCR**. The significantly differentially regulated genes detected by the microarray were randomly selected for real-time RT-PCR analysis. Correlation analysis of the results between real time RT-PCR and cDNA microarray were calculated (*P* < 0.05).

To study the mechanism of sensitivity to ABA mediated by *ThbZIP1* at the genome scale, cDNA microarray analysis was performed to compare the gene expression between Arabidopsis overexpressing ThbZIP1 and WT under ABA treatment condition. There were 1662 and 1609 genes significantly upregulated or downregulated, respectively, by the overexpression of ThbZIP1 under ABA treatment condition (Supplementary Data S1, Supplementary Table S1). To determine the validity of the microarray results, twelve differentially expressed genes identified by the microarray were randomly selected for real-time RT-PCR analyses. The results showed high correlation coefficients between the real-time PCR and microarray data (*R*^2^ = 0.9774, *P* < 0.05) (Figure 5), which validated the reliability of the microarray results.

#### GO classification of the genes differentially regulated under ABA stress

**Figure 6:**
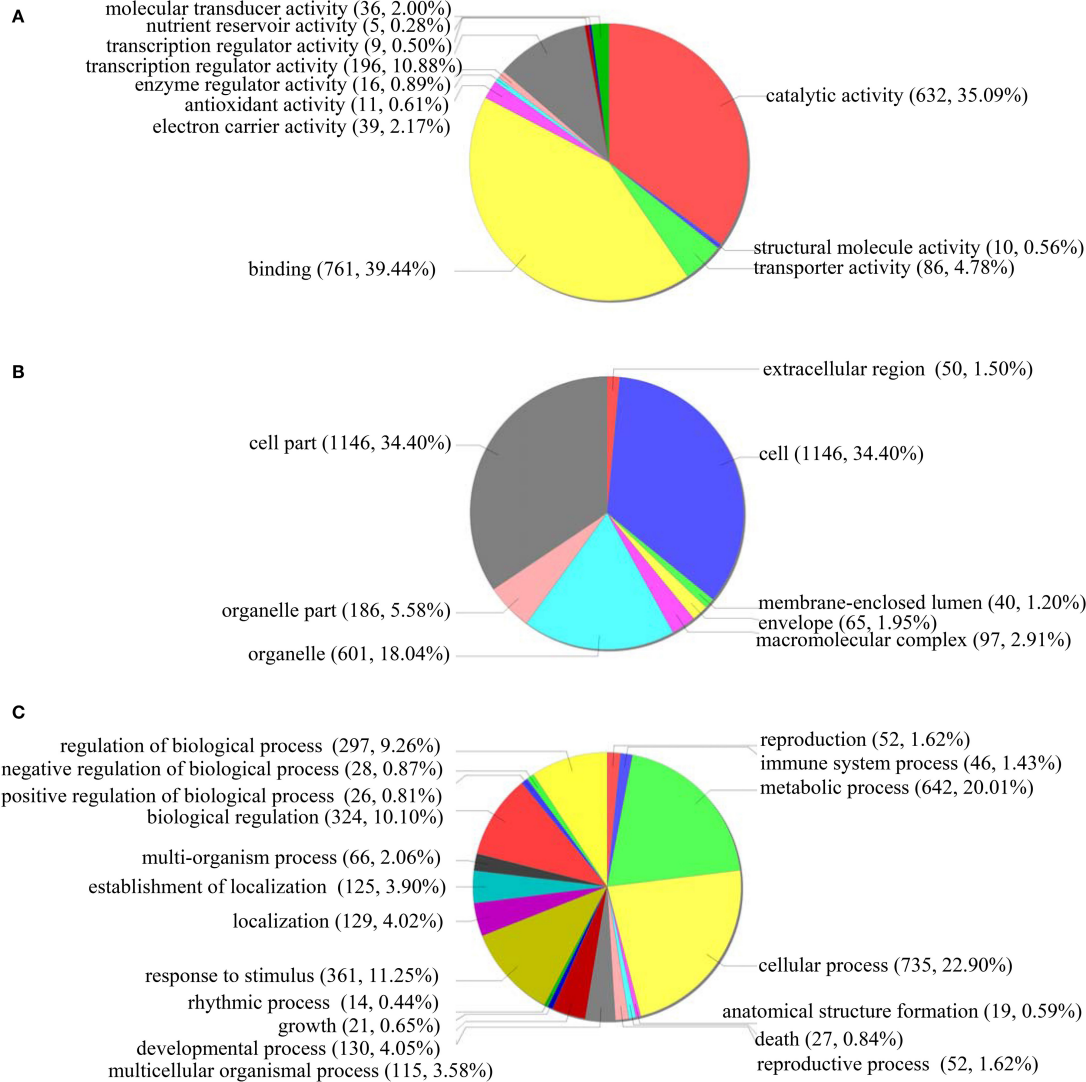
**GO term pie chart of the gene differentially regulated by ABA stress. (A–C)** indicates GO term pie picture of “Molecular function,” “Cellular component,” and “Biological process” for the gene differentially regulated under ABA stress, respectively.

GO classification was conducted for the functions significantly (*p* < 0.05) enriched under ABA treatment condition. In the “Molecular function” term, the most highly enriched terms were “Catalytic activity” (632 genes), “Binding function” (761 genes) and “Transcription regulator activity factor” (196 genes) (Figure 6A). For the GO term “Cellular component,” 1146 genes were enriched in “Cell” and “Cell part” component genes, while 601 and 186 genes belong to “Organelle” and “Organelle part,” respectively (Figure 6B). In the “Biological process” term, the differentially regulated genes were enriched in the many GO categories, such as “Cellular process” (735 genes), “Metabolic process” (642 genes), “Response to stimulus” (361 genes), “Biological regulation” (324 genes), “Regulation of biological process” (297 genes), and “Developmental process” (130 genes) (Figure 6C). These results indicated that under ABA treatment conditions, many biological pathways were altered, which should contribute to the sensitivity to ABA.

In “Molecular function” term, the downregulated genes in the “Transcription regulator activity factor” accounted for 60%, demonstrating that transcriptional activity decreased when exposed to ABA treatment. In the “Cellular component” term, 79.03% of genes involved in “Organelle part” were upregulated, suggesting that organelle parts are increased under ABA treatment. However, in “Biological process” term, 55.12% of genes involved in “Response to stimulus” were downregulated, indicating that the genes' responses to stimuli were mainly inhibited by ABA. In addition, the downregulated genes in “Biological regulation” and “Regulation of biological process,” respectively, accounted for 59.26% and 60.27%, suggesting that the activities of “biological regulation” and “regulation of biological process” were inhibited under ABA treatment. Therefore, the pathways associated with these decreased or increased functions may lead to the ABA sensitivity of plants overexpressing ThbZIP1.

#### Target genes regulated by ThbZIP1 in response to ABA

Previously, our studies showed that ThbZIP1 can specifically bind to ACGT elements, including A-box (“TACGTA”), C-box (“CACGTC”) and G-box (“CACGTG”), but with different binding affinities (Ji et al., 2013). In the present study, to study whether ThbZIP1 regulates the expression of genes *via* binding to the C-, G- or A-box motifs when exposed to ABA treatment, we randomly selected the genes upregulated by ThbZIP1 under ABA stress condition, and screened their promoter regions (−1 to 1000 bp) for C-, G- or A-box motifs. The results showed that all of the selected genes have at least one of C-box, G-box or A-box in their promoter region, and most of them have two or more C-, G- or A-box motifs (Table 1). This result suggested that ThbZIP1 regulates these genes *via* binding to C-, G- or A-box motifs present in their promoter sequences when exposed to ABA.

**Table 1:** **The distribution of C-, G, or A-box in the promoter of the ThbZIP1 target genes under ABA treatment condition**.

**Locus tag**	**Fold change**	**bZIP recognition sequences**	**Description**
AT3G17520	81.7964	−231 (CACGTG)	Protein coding
AT2G21660	19.792	−157 (CACGTG)	Glycine-rich RNA-binding protein 7
AT3G46640	19.0602	−245 (CACGTG)	Protein phytoclock 1
AT4G30650	10.3677	−271 (CACGTG)	Putative low temperature and salt responsive protein
AT3G11020	8.0937	−787 (CACGTG)	Dehydration-responsive element-binding protein 2B
AT2G38465	7.5216	−310 (CACGTG)	Hypothetical protein
		−496 (GACGTC)	
AT5G15800	7.2404	−482 (CACGTG)	Developmental protein SEPALLATA 1
AT5G59570	7.2356	−91 (CACGTG)	myb Family transcription factor
		−544 (TACGTA)	
AT1G78440	7.2322	−322 (GACGTC)	Gibberellin 2-beta-dioxygenase 1
AT4G27654	7.2027	−306 (TACGTA)	Hypothetical protein
AT5G57110	7.1898	−125 (CACGTG)	Calcium-transporting ATPase 8
AT1G68050	6.7293	−666 (TACGTA)	Adagio protein 3
AT2G36390	5.7195	−201 (CACGTG)	1,4-alpha-glucan branching enzyme
AT5G19340	5.5723	−766 (TACGTA)	Hypothetical protein
AT1G16850	5.4176	−134 (CACGTG)	Hypothetical protein
AT1G79440	5.3763	−207 (CACGTG)	Succinate-semialdehyde dehydrogenase
AT3G10410	5.3618	−88 (CACGTG)	Carboxypeptidase
		−464 (CACGTG)	
		−841 (CACGTG)	
		−869 (CACGTG)	
		−879 (CACGTG)	
AT1G10760	5.3329	−533 (CACGTG)	Alpha-glucan water dikinase
		−579 (CACGTG)	1
AT5G57785	5.2354	−196 (CACGTG)	Hypothetical protein
AT4G09020	4.9709	−148 (CACGTG)	Isoamylase 3
AT1G48330	4.9437	−286 (CACGTG)	Hypothetical protein
		−413 (TACGTA)	
		−828 (TACGTA)	
AT5G14550	4.5091	−297 (CACGTG)	Core-2/I-branching
		−592 (CACGTG)	beta-1,6-N- acetylglucos-aminyltransferase family protein
AT3G15950	4.384	−129 (CACGTG)	DNA topoisomerase like protein
AT5G64860	4.1671	−115 (CACGTG)	4-alpha-glucanotransferase-like protein
		−126 (CACGTG)	
AT1G09350	4.1117	−345 (CACGTG)	Galactinol synthase 3
AT2G43550	4.0556	−182 (CACGTG)	Defensin-like protein 197
AT3G05880	4.0005	−498 (TACGTA)	Hydrophobic protein RCI2A
		−956 (CACGTG)	
AT3G12970	3.9478	−150 (CACGTG)	Hypothetical protein
AT1G15830	3.9475	−438 (GACGTC)	Hypothetical protein
		−727 (CACGTG)	
AT5G05410	3.8916	−979 (TACGTA)	Dehydration-responsive element-binding protein 2A
AT5G18540	3.8882	−239 (GACGTC)	Hypothetical protein
AT4G03210	3.8147	−667 (GACGTC)	Xyloglucan endotransglucosylase/hydrolase protein 9
AT2G42540	3.7073	−252 (CACGTG)	Cold-regulated protein 15a
		−428 (CACGTG)	

#### Comparison of gene expression profiles between salt and ABA treatments

Our previous studies showed that the *ThbZIP1* transformed tobacco and Arabidopsis plants displayed improved salt and drought stress tolerance. Meanwhile, the *ThbZIP1* transformed plants displayed enhanced activities of superoxide dismutase (SOD), peroxidase (POD), and glutathione S-transferase (GST); however, reactive oxygen species level, electrolyte leakage rate, and malondialdehyde (MDA) content were all significantly decreased in *ThbZIP1* transformed plants under salt and drought stress condition (Wang et al., 2010; Ji et al., 2013). These results suggested that ThbZIP1 confers salt and drought tolerance to plants by decreasing ROS injury and protect cell membrane from damage. However, although *ThbZIP1* transformed plants showed increased sensitivity to ABA, the ROS level, and electrolyte leakage rate in *ThbZIP1* transformed plants were also significantly lower than in WT plants under ABA treatment condition (Ji et al., 2013). Consistently, Zhang et al. (2012) showed that a maize *bZIP* gene, *ABP9*, confers salt, drought, and cold tolerance to transgenic plants, but plants overexpression of *ABP9* also displayed significantly enhanced sensitivity to ABA. The transgenic plants overexpressing *ABP9* was found to improve salt stress tolerance by decreasing ROS accumulation level; however, it also displayed decreased ROS accumulation under ABA treatment conditions. In addition, *bZIP* overexpressing plants can reduce stomatal aperture when treated with ABA, and both *ABP9* and *ThbZIP1* transformed plants showed improved water-conserving capacity (Zhang et al., 2012; Ji et al., 2013). ROS injury, damage of cell membrane (reflected by and electrolyte leakage rate) and decreased water-conserving capacity are the main factors that damage plants under abiotic stress conditions. However, exogenously applied ABA damaged the plants overexpressing *ThbZIP1* obviously not through the pathways of ROS injury, damage of cell membrane, and decreased water-conserving capacity. Therefore, it is deserved to discover the mechanism of sensitivity to ABA mediated by *ThbZIP1*, and comparing the expression of genes in *ThbZIP1* plants in response to salt and ABA can be helpful in revealing this mechanism.

**Figure 7:**
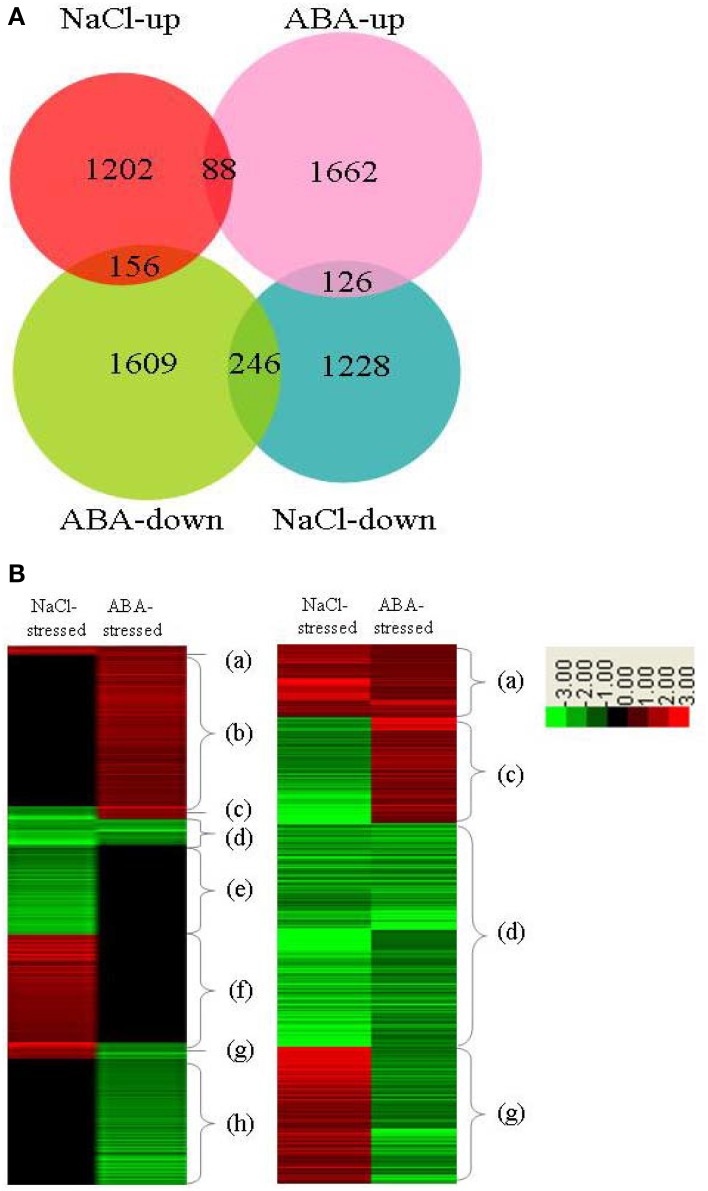
**Analysis of the distribution of genes differentially regulated by ThbZIP1 in response to ABA and salt**. **(A)** Venn diagram for the distribution of genes differentially regulated in response to ABA and salt. **(B)** Hierarchical analysis of the genes differentially regulated by ThbZIP1 under salt or ABA stimulus. Red means induced expression, green indicates decreased expression and black shows unchanged expression.

In previous investigation, we studied genes whose expressions were regulated by *ThbZIP1* under salt stress using a cDNA microarray (Ji et al., 2013). Therefore, we compared the expression of genes in *ThbZIP1* transformed plants in response to salt and ABA. The results showed that ABA treatment differentially regulates more genes than salt stress (Figure 7). There were 88 and 246 genes that were upregulated and downregulated, respectively, in both NaCl- and ABA-stressed plants. There were 156 genes upregulated by NaCl treatment but downregulated by ABA treatment, and 126 genes that were downregulated by NaCl treatment but upregulated by ABA treatment (Figure 7A). Genes differentially regulated by both salt and ABA treatment only accounted for a small proportion (9.75%), suggesting that the responses of ThbZIP overexpressing plants to ABA and salt stress are quite different (Figure 7A). Hierarchical cluster analysis was then performed on the genes differentially regulated by ABA or salt. The genes were grouped into eight groups (a–h) according to their expression patterns (Figure 7B). The results showed that groups a, b, e, and f accounted for more than 90% of the differentially regulated genes, and these groups represented the genes that were only differentially expressed by ABA or salt, but were unaffected by the other treatment (Figure 7B). Such a high percentage of genes that showed different expression patterns in response to salt and ABA stimulus further suggested that the response to salt and ABA mediated by ThbZIP1 are quite different. Thus, the genes that only are differentially regulated in salt or ABA treatment contribute to the different salt and ABA responses.

GO analysis was conducted to study the similarity and difference in biological process mediated by ThbZIP1 between salt and ABA stimuli. The results showed that under ABA treatment, the upregulated genes were significantly enriched in 12 subgroups, and the downregulated genes were significantly enriched in six subgroups (Table 2). However, under salt stress conditions, the upregulated genes were enriched in four subgroups and the downregulated genes were enriched in eight subgroups (Table 2). Importantly, among these enriched GO terms, six terms that were induced by ABA were decreased by salt; meanwhile, two subterms that were decreased by ABA were induced by salt (Table 2). These results suggested that structural molecule activity, organelle part, membrane-enclosed lumen, reproduction, reproductive process, and organelle are enhanced by ABA treatment but inhibited by salt stress. Conversely, immune system process and multi-organism process were improved by salt stress but inhibited by ABA. Under ABA treatment, the pathways of transcription regulator activity, enzyme regulator activity, and developmental process were significantly altered; however, these pathways were not affected by salt stress. Furthermore, the up- and downregulated genes were both enriched in metabolic process under salt stress conditions; however, metabolic process were only enriched by the genes downregulated by ABA stimuli, indicating that metabolic process should be repressed. Therefore, the genes associated with these differences in the response to salt and ABA should be involved in salt tolerance and ABA sensitivity.

**Table 2:** **The significantly enriched GO terms of the differentially regulated genes under ABA and salt treatment condition**.

**GOId**	**Name**	**Percent (%)**	**The number of up or down-regulated genes**	**The number of differentially regulated genes**
**UNDER ABA TREATMENT CONDITION (UP-REGULATED GENES)**				
GO:0031975	Envelope	93.85	61	65
GO:0045182	Transcription regulator activity	88.89	8	9
GO:0010926	Anatomical structure formation	84.21	16	19
GO:0005198	Structural molecule activity	80.00	8	10
GO:0044422	Organelle part	79.03	147	186
GO:0031974	Membrane-enclosed lumen	70.00	28	40
GO:0000003	Reproduction	65.38	34	52
GO:0022414	Reproductive process	65.38	34	52
GO:0048511	Rhythmic process	64.29	9	14
GO:0043226	Organelle	61.06	367	601
GO:0032991	Macromolecular complex	60.82	59	97
GO:0005576	Extracellular region	60.00	30	50
**UNDER ABA TREATMENT CONDITION (DOWN-REGULATED GENES)**				
GO:0002376	Immune system process	80.43	37	46
GO:0051704	Multi-organism process	68.18	45	66
GO:0016265	Death	66.67	18	27
GO:0016209	Antioxidant activity	63.64	7	11
GO:0030528	Transcription regulator activity	61.73	121	196
GO:0050789	Regulation of biological process	60.27	179	297
**UNDER SALT TREATMENT CONDITION (UP-REGULATED GENES)**				
GO:0009055	Electron carrier activity	61.11	11	18
GO:0005215	Transporter activity	60.87	14	23
GO:0051704	Multi-organism process	60.00	12	20
GO:0002376	Immune system process	75.00	9	12
**UNDER SALT TREATMENT CONDITION (DOWN-REGULATED GENES)**				
GO:0005198	Structural molecule activity	66.67	6	9
GO:0044422	Organelle part	65.79	25	38
GO:0031974	Membrane-enclosed lumen	75.00	6	8
GO:0043226	Organelle	60.31	79	131
GO:0009893	Positive regulation of metabolic process	75.00	6	8
GO:0032501	Multicellular organismal process	62.07	18	29
GO:0000003	Reproduction	68.75	11	16
GO:0022414	Reproductive process	73.33	11	15

GO analysis was further performed on the 88 genes that were up-regulated by both ABA and salt treatment, and 49, 53, and 55 genes were respectively classified into the terms of molecular function, cell component, and biological process (Supplementary Data S2). According to GO analysis in biological process, there were 27 out of 55 genes having the specific function involved in the response of abiotic stress or defense. Consideration of these genes were up-regulated by ABA treatment, this result indicated that these genes might play their roles in abiotic stress or defense response through ABA signaling pathway. Furthermore, molecular function classification showed that a high percentage of genes (69.4%) are involved in binding function, such as nucleotide binding, ATP binding, and ion binding, suggesting that these functions play important roles in both ABA and salt response.

## Conclusions

In the present study, based on the ABRE motifs present in the promoter of ThbZIP1, we identified that the TF ThABF1 directly regulates the expression of ThbZIP1. The mechanism of ABA sensitivity mediated by ThbZIP1 was studied on a genome scale, and processes regulated by ThbZIP1 in response to ABA were revealed. Plants overexpressing ThbZIP1 are tolerant to salt but sensitive to ABA; therefore, we further studied these differences on a genome scale. The results showed that some processes were highly altered in *ThZIP1* overexpressing plants when exposed to salt or ABA treatment, and these dramatically changed biological processes should be contributed to the tolerance to salt but sensitivity to ABA.

### Conflict of interest statement

The authors declare that the research was conducted in the absence of any commercial or financial relationships that could be construed as a potential conflict of interest.
