# Supplementary material for: The regulatory network of ThbZIP1 in response to abscisic acid treatment
Source: Front Plant Sci. 2015 Feb 10;6:25. doi: 10.3389/fpls.2015.00025 (PMC4322638; doi:10.3389/fpls.2015.00025)
Supplement: Supplementary file 3 [file DataSheet3.DOC]

***Supplementary Material***

**The function and regulatory network of ThbZIP1 in response to abscisic acid treatment**

**Xiaoyu Ji ^1, †^, Guifeng Liu ^2, †^, Yujia Liu ^3^, Xianguang Nie ^2^ , Lei Zheng ^2^, Yucheng Wang ^1^***

^1^ Key Laboratory of Biogeography and Bioresource in Arid Land, Xinjiang Institute of Ecology and Geography, Chinese Academy of Sciences, Urumqi, Xinjiang China

^2^ State Key Laboratory of Tree Genetics and Breeding (Northeast Forestry University), Harbin, China

^3^ College of Food Engineering, Harbin University of Commerce, Harbin University of Commerce, Harbin, China

^†^ These authors contributed equally to this work

*** Correspondence:** Yucheng Wang, Key Laboratory of Biogeography and Bioresource in Arid Land, Xinjiang Institute of Ecology and Geography, Chinese Academy of Sciences, Urumqi, 830011, Xinjiang, China.

[wangyucheng@ms.xjb.ac.cn](mailto:wangyucheng@ms.xjb.ac.cn)

1. **Supplementary Data**

**Supplementary Data S1. Significantly differentially regulated genes (p<0.05; ratio>2 or <0.5) in *ThbZIP1* transformed Arabidopsis plants relative to WT Arabidopsis plants under ABA stress conditions.**

**Supplementary Data S2. Gene ontology analysis of the 88 genes that were up-regulated by both ABA and salt treatment conditions.**

1. **Supplementary Figures and Tables**

## Suplementary Tables

Supplemental Table S1. Genes highly enriched in ThbZIP1 overexpressed plants under ABA stress conditions.

| **Gene name** | **Number** | **Locus tag** |
| --- | --- | --- |
| Zinc finger family protein | 16 | AT3G07650 AT1G28050 AT2G47890 AT5G48250 AT5G15840 AT1G24625 AT5G25160 AT1G69600 AT2G47850 AT3G27330 AT5G60250 AT5G60710 AT2G29660 AT4G16610 AT3G49930 AT5G10970 |
| MYB | 10 | AT2G38090 AT5G59570 AT1G49560 AT5G07700 AT5G11510 AT5G60890 AT3G61250 AT2G31180 AT3G01140 AT3G12820 |
| ERF | 16 | AT4G20880 AT5G25810 AT5G61890 AT1G64380 AT2G20880 AT1G77200 AT2G44940 AT4G31060 AT2G23340 AT4G23750 AT4G11140 AT5G44350 AT1G16060 AT5G60120 AT3G54990 AT4G37750 |
| WRKY | 7 | AT4G31805 AT2G40750 AT2G03340 AT4G24240 AT2G34830 AT4G39410 AT2G44745 |
| bHLH | 7 | AT5G39860 AT1G22490 AT5G46690 AT5G51780 AT3G05800 AT4G00870 AT3G20640 |
| Cytochrome P450 | 15 | AT5G52320 AT1G63710 AT1G01600 AT4G37410 AT1G13710 AT1G11600 AT3G10570 AT1G19630 AT2G34490 AT1G78490 AT4G12320 AT5G44620 AT4G13770 AT2G45560 AT3G48290 |
| Heat shock protein | 10 | AT3G12580 AT5G56010 AT5G56030 AT4G17750 AT5G55570 AT2G40130 AT5G03720 AT2G03020 AT5G56010 AT5G52640 AT3G12580 AT3G53800 AT2G20550 AT2G19310 AT5G23240 |
| Protein kinase family | 9 | AT1G70430 AT2G18890 AT5G61570 AT3G59350 AT1G09450 AT1G50240 AT5G18700 AT2G31010 AT2G28930 |
| Glycosyl hydrolase family protein | 7 | AT5G64710 AT3G61100 AT3G62460 AT3G07320 AT5G55180 AT1G11820 AT2G05790 |
| Auxin-responsi-ve protein | 9 | AT4G12410 AT3G09870 AT3G12955 AT5G35735 AT4G17280 AT4G29080 AT4G14550 AT1G19850 AT2G33860 |
| Growth-regulating factor | 4 | AT2G45480 AT5G53660 AT3G52910 AT2G22840 |
| Arabinogalacta-n protein | 11 | AT2G14890 AT5G65390 AT5G24105 AT1G28290 AT3G06360 AT2G47930 AT5G18690 AT1G55330 AT5G11740 AT4G26320 AT5G64310 |
| Aspartyl protease family protein | 8 | AT1G01300 AT1G09750 AT2G03200 AT4G33490 AT3G12700 AT3G25700 AT5G07030 AT5G02190 |
| Lipid-transfer protein | 8 | AT4G15160 AT1G62500 AT4G00165 AT1G62510 AT3G53980 AT4G33550 AT1G48750 AT3G22142 |
| Cell cycle protein | 9 | AT4G24010 AT2G21770 AT1G79560 AT4G33270 AT4G33260 AT4G22860 AT4G11990 AT3G05480 AT1G49910 |
| Cyclin | 24 | AT2G44740 AT2G45080 AT1G27630 AT1G73690 AT1G20930 AT1G76540 AT2G38620 AT3G54180 AT4G34160 AT1G76310 AT1G20610 AT4G35620 AT2G17620 AT2G26760 AT3G11520 AT5G06150 AT4G37490 AT1G80370 AT1G15570 AT5G11300 AT1G44110 AT3G05330 AT1G16330 AT1G34460 |
| Dehydration-responsive element-binding protein | 6 | AT5G11590 AT3G11020 AT5G05410 AT4G25470 AT4G25490 AT4G25480 |
| Early nodulin-like protein | 7 | AT1G64640 AT5G25090 AT4G27520 AT4G12880 AT1G08500 AT4G31840 AT2G25060 |
| GDSL esterase | 12 | AT5G33370 AT1G54020 AT4G26790 AT4G01130 AT4G28780 AT5G45670 AT5G41890 AT1G29670 AT1G54010 AT5G45960 AT1G28610 AT5G14450 |
| Glycine-rich protein | 10 | AT1G64450 AT4G39260 AT2G21660 AT3G23830 AT4G13850 AT2G05510 AT2G05540 AT5G61660 AT2G36120 AT4G18280 |
| Homeobox protein | 11 | AT1G73360 AT5G15150 AT1G79840 AT1G70920 AT5G66700 AT2G18550 AT1G62360 AT1G23380 AT1G75240 AT3G50890 AT5G39760 |
| Plant invertase | 7 | AT5G62360 AT5G20740 AT1G23205 AT1G62770 AT1G23350 AT2G47670 AT3G47380 |

Supplemental Table S2. Primer sequences used in the yeast one-hybrid analyses.

| **Primer names** | **Primers sequences (**5’-3’**)** |
| --- | --- |
| pHIS2-AF | AATTCCAACGTGCCCAACGTGCCCAACGTGCCGAGCT |
| pHIS2-AR | CGGCACGTTGGGCACGTTGGGCACGTTGG |
| pHIS2-AM1F | AATTCCCCCGTGCCCCCCGTGCCCCCCGTGCCGAGCT |
| pHIS2-AM1R | CGGCACGGGGGGCACGGGGGGCACGGGGG |
| pHIS2-AM2F | AATTCCAACGCAACCAACGCAACCAACGCAACGAGCT |
| pHIS2-AM2R | CGTTGCGTTGGTTGCGTTGGTTGCGTTGG |
| pHIS2-AM3F | AATTCCCCAACAACCCCAACAACCCCAACAACGAGCT |
| pHIS2-AM3R | CGTTGTTGGGGTTGTTGGGGTTGTTGGGG |
| pHIS2-ABREp+F | CCGGAATTCAGACGACCAACGTGCGCCAACC |
| pHIS2-ABREp+R | CGAGCTCCTATCTCAGAAGGCACGTTGTT |
| pHIS2-ABREp-F | CCGGAATTCCTTTGACGATTCTCACCAAACC |
| pHIS2-ABREp-R | CGAGCTCCCGTTTGTTCTATCGTTAGATA |
| *ThABF1-Rec2*F | AAGCAGTGGTATCAACGCAGAGTGGCCATTATGGCCC ATGGAGGCTTCAACAATGGGAC |
| *ThABF1-*Rec2R | TCTAGAGGCCGAGGCGGCCGACATGGGTGGTGGGTG  GTGGTGCAGCA |

Supplemental Table S3. Primer sequences used for construction of the reporter and effector vectors.

| **Primer names** | **Primers sequences (**5’-3’**)** |
| --- | --- |
| pCAM-ABREF | AGCTTCAACGTGCCCAACGTGCCCAACGTGCCACCCTTCCTCTATATAAGGAAGTTCATTTCATTTGGAGAGAACACGGC |
| pCAM-ABRER | CATGGCCGTGTTCTCTCCAAATGAAATGAACTTCCTTATATAGAGGAAGGGTGGCACGTTGGGCACGTTGGGCACGTTGA |
| pCAM-mABREF | AGCTTCCCAACAACCCCAACAACCCCAACAACACCCTTCCTCTATATAAGGAAGTTCATTTCATTTGGAGAGAACACGGC |
| pCAM-mABRER | CATGGCCGTGTTCTCTCCAAATGAAATGAACTTCCTTATATAGAGGAAGGGTGTTGTTGGGGTTGTTGGGGTTGTTGGGA |
| pCAM-ABREp+F | AGCTTAGACGACCAACGTGCGCCAACCACCCTTCCTCTATATAAGGAAGTTCATTTCATTTGGAGAGAACACGGC |
| pCAM-ABREp+R | CATGGCCGTGTTCTCTCCAAATGAAATGAACTTCCTTATATAGAGGAAGGGTCCTATCTCAGAAGGCACGTTGTTA |
| pCAM-ABREp-F | AGCTTCTTTGACGATTCTCACCAAACCACCCTTCCTCTATATAAGGAAGTTCATTTCATTTGGAGAGAACACGGC |
| pCAM-ABREp-R | CATGGCCGTGTTCTCTCCAAATGAAATGAACTTCCTTATATAGAGGAAGGGTCCCGTTTGTTCTATCGTTAGATAA |
| pROKII-ThABF1F | CTCTAGAGGATCCCCATGGAGGCTTCAACAATGGGAC |
| pROKII-ThABF1R | TCGAGCTCGGTACCCTCAGGTGGTGGGTGGTGGTGC |

Supplemental Table S4. Primer sequences used for the ChIP assay.

| **Primer names** | **Primers sequences (**5’-3’**)** |
| --- | --- |
| GFP-ThABF1F | CTCTAGAGGATCCCCATGGAGGCTTCAACAATGGGACCT |
| GFP-ThABF1R | GGTGGTGGGTGGTGGTGCAGCAG |
| GFPR | TCGAGCTCGGTACCCTCACTTGTACAGCTCATCCATGCC |
| ChIP-1F | CTCACTAAACAATTTGATGT |
| ChIP-1R | ATCATGAATTGAAATAATCC |
| ChIP-2F | CGATTAATTAGTTTAATTATC |
| ChIP-2R | CATTTTTGTATCATTTTATTAC |
| ChIP-3F | GTAATAAAATGATACAAAAATG |
| ChIP-3R | TGAAGAGATAAGGGGTATTG |
| ChIP-4F | TATTGCACGCCAGCTGTAAC |
| ChIP-4R | ACTAAAATCATTTGCTCAAT |
| ActinF | AAACAATGGCTGATGCTG |
| ActinR | ACAATACCGTGCTCAATAGG |

Supplemental Table S5: Primer sequences used in real time RT-PCR.

| **Gene** | **GenBank Accession number** | **Forward primers (5'-3')** | **Reverse primers (5'-3')** |
| --- | --- | --- | --- |
| *ThbZIP1* | Fj752700 | TGTTCGCTACGCAAACTTGGAC | TAATTCCTCTACGTCAGCATTC |
| *ThABF1* | JX169810 | CCTACCTTGGGCGAGATGACCT | GGTGCCAGTGGCAATTGATTCT |
| *Actin* | FJ618517 | AAACAATGGCTGATGCTG | ACAATACCGTGCTCAATAGG |
| *α-tubulin* | FJ618518 | CACCCACCGTTGTTCCAG | ACCGTCGTCATCTTCACC |
| *β-tubulin* | FJ618519 | GGAAGCCATAGAAAGACC | CAACAAATGTGGGATGCT |

Supplemental Table S6: Primer sequences used in real time RT-PCR for verification of microarray data.

| **Gene** | **DGE** | **Forward primers (5'-3')** | **Reverse primers (5'-3')** |
| --- | --- | --- | --- |
| AT5G48250 | 13.7663 | GGAAATCTCATTTCAGATGAAG | TGAAAGCTGCTTCATCGATGAC |
| AT2G42800 | 10.333 | AGGAATCTCGAACCTTCAGAGT | GGAACAACGCCGATCAGTAAGT |
| AT3G22830 | 8.1717 | AACAAATGATGAGCTTCCTTGC | AGCAAGTTTGTCCAACTCCGAC |
| AT1G52030 | 6.3136 | CTTATGTGAAGTTCGAATACAG | GAACATCACTAGCTTTCCCATG |
| AT4G26790 | 4.7033 | TCGTGACAGATATATACAGACT | AAACCGAATGCTTCAGGATGGT |
| AT1G10780 | 3.8936 | GCAGCAAGCAAGAGACGTGAAG | CACTAGTGATTCAAGCGTGTTC |
| AT4G00970 | 0.4887 | AGTTGCAGGAACCTACGGTTAC | ACAACAGAAGCCATTGTTGGTC |
| AT5G67030 | 0.4138 | ACTGGGTCCTTGGAGGTAACAG | CGAAACCTGAGACGAAGGGATC |
| AT4G01540 | 0.391 | GAATGACTTGAGAATCCCAATG | AGATATGGATCCAGCATCACTG |
| AT5G67340 | 0.3118 | TTCAGCTTGTCGGTGATCGAAG | ACTCCACAACTTCCACCAATAC |
| AT2G41640 | 0.2959 | ATTGGGAATCAAACCATTGTTG | ATGAGTCATTGCTGCTCCATGT |
| AT2G43000 | 0.2604 | CGATTGGATGATGCATGAATTC | CCAACTATATGTTGGTTCCAAT |
| *Actin* |  | AAACAATGGCTGATGCTG | ACAATACCGTGCTCAATAGG |
| *α-tubulin* |  | CACCCACCGTTGTTCCAG | ACCGTCGTCATCTTCACC |
| *β-tubulin* |  | GGAAGCCATAGAAAGACC | CAACAAATGTGGGATGCT |

## Suplementary Figures


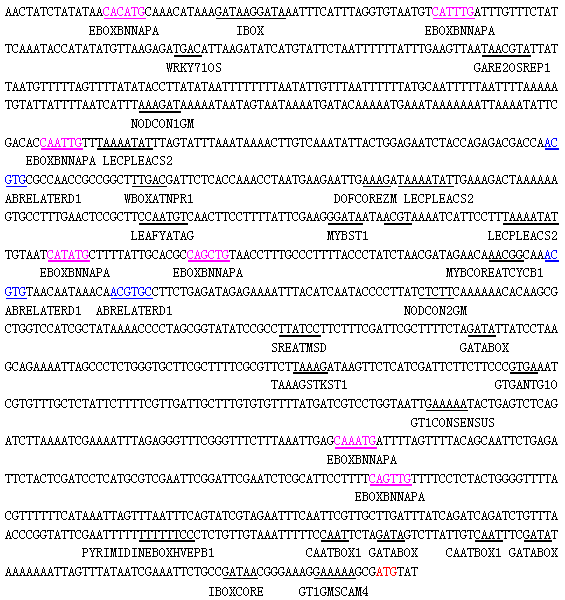


Supplementary Figure 1. Promoter activity assay of *ThbZIP1*.
